# Limitations to clinically restoring meaningful peripheral nerve function across gaps and overcoming them

**DOI:** 10.3389/ebm.2025.10566

**Published:** 2025-05-27

**Authors:** Christian A. Foy, Damien P. Kuffler

**Affiliations:** ^1^ Section of Orthopedic Surgery, University of Puerto Rico, San Juan, PR, United States; ^2^ Institute of Neurobiology, Medical School, University of Puerto Rico, San Juan, PR, United States

**Keywords:** allograft, axon regeneration, collagen tube, nerve gap, nerve trauma, peripheral nerve repair, platelet-rich fibrin, PRP

## Abstract

Clinically, reliably restoring meaningful peripheral sensory and motor nerve function across peripheral nerve gaps is limited. Thus, although autografts are the clinical “gold standard” repair technique for bridging nerve gaps, even under relatively good conditions, <50% of patients recover meaningful function. Due to this low recovery rate, many patients are not even provided repair surgery and, consequently, suffer permanent loss of function. This paper examines intrinsic and extrinsic changes associated with injured neurons and Schwann cells that reduce the extent of axon regeneration and recovery. It also examines how these changes can be reversed, leading to enhanced regeneration and recovery. It next examines the efficacy of platelet-rich plasma (PRP) in promoting axon regeneration and two novel techniques involving bridging nerve gaps with an autograft within a platelet-rich (PRP) collagen tube or only a PRP-filled collagen tube, which induce meaningful recovery under conditions where autografts alone are not effective. Finally, it looks at potential mechanisms by which platelet-released factors may enhance axon regeneration and recovery. This review shows that although there are many limitations to restoring meaningful function following peripheral nerve trauma, there are a number of ways these can be overcome. Presently, the most promising techniques involve using PRP.

## Impact statement

Restoring clinical function following peripheral nerve trauma is restricted by neuron and Schwann cell intrinsic and extrinsic limitations. Further, autografts, the current clinical “gold stand” technique for bridging nerve gaps to restore function, suffer many significant limitations in restoring meaningful functional recovery. This review discusses intrinsic and intrinsic limitations to regeneration and how they can be overcome. It also discusses how the application of platelet-rich plasma (PRP) promotes axon regeneration and how its influences can be increased or decreased. It then discusses how, clinically, bridging nerve gaps with autograft within a PRP-filled collagen tube induces axon regeneration and recovery under currently impossible conditions. It concludes with a discussion of the potential mechanisms by which platelet-released factors may exert their influences. Understanding what limits axon regeneration and recovery and how these limitations can be overcome will lead to developing new clinical techniques that induce more extensive axon regeneration and recovery.

## Introduction

Sensory nerve autografts, the clinical “gold standard” technique for restoring function across peripheral nerve gaps [1], have substantial limitations. Therefore, there is a good prognosis for reliable, meaningful sensory and motor function only when (1) the repairs are performed ≤5 months post-trauma [2–4], with recovery decreasing with longer delays [3–6] (2) the gaps are <5 (cm) [7, 8], with recovery decreasing for longer gaps [2, 3, 8, 9], few axons regenerate across grafts ≥8 cm in length [2, 10, 11], and none across autografts >10 cm [3, 5], and (3) patients are ≤20–25 years old, with recovery decreasing with increasing ages [3, 4, 6]. Finally, there is little to no recovery when the values of two or all three variables increase simultaneously [9, 12]. Therefore, <50% of subjects recover meaningful sensory or motor functions [13]. These findings raise the question of what underlies these limitations and how can they are reduced, leading to improved recovery.

## Injury-induced intrinsic neuronal changes reduce their capacity to extend axons

Partly underlying the decreased capacity of aged and long-term axotomized neurons to extend axons are changes in their intrinsic properties [14]. These neurons lose their capacity to extend axons, and those extended regenerate only short distances [15, 16] while regenerating more slowly than normal [17]. Thus, by >4 months post-nerve injury, only about 33% of neurons can extend an axon [15, 18], and for those that retain the capacity, it is reduced to ˂10% of normal [16].

### Reduced protein synthesis

The c-Jun transcription factor is critical for neurons’ capacity to extend axons, and nerve injury induces neuronal up-regulation of c-Jun expression. However, with increasing time of axotomy, c-Jun expression decreases, paralleling the loss of neurons’ capacity to extend axons [19]. This change is also associated with the down-regulation of genes for regeneration-promoting neurotrophic factors, such as GAP-43 and α1-tubulin [20], NGF [21], BDNF, and CNTF [16, 22]. Thus, the age-associated decrease in axon regeneration is due to reduced protein synthesis, which is required to induce the neurons’ soma to respond to injury by triggering the regeneration process and growth cone extension [23–25]. This process also involves decreased levels of axonal translation proteins and the inability of neurons to increase the translation of regeneration-promoting axonal mRNAs released from stress granules [26]. The decrease is also associated with an increasing age-associated decrease in neurofilament mRNA levels and neurofilament proteins [27], and the loss of Nrg1, which reduces axon-Schwann cell interactions and remyelination after nerve crush, further reducing neurons’ capacity to extend axons [28].

### Decreased metabolism and axoplasmic transport

Neurite outgrowth from neonatal neurons *in vitro* is 40% faster than adult neurons [29]. This is attributed to an age-related decrease in cytoskeletal protein expression [30] and axoplasmic transport, which are required for axon elongation [30, 31]. This is because axon regeneration requires energy metabolism, which involves oxidative glycolysis and the formation of high-energy phosphate compounds, most importantly creatine phosphate and ATP [32]. Increasing age is also associated with a decrease in the levels of endoneurial ATP and creatine phosphate [30], which would, therefore, restrict the extent of axon regeneration.

## Reversing injury-induced intrinsic neuronal changes allows neurons to extend axons by promoting neuron protein synthesis

Axon regeneration following a sciatic nerve crush is promoted by enhanced protein synthesis due to enhanced local translation and production of the protein synthesis machinery [26]. This involves dissolving stress granules, resulting in their releasing sequestered mRNAs and translation factors [33]. Further, following rat sciatic nerve injury, Nrg1 treatment increases axon diameter, myelin thickness, distance axons regenerate, and both the extent [34] and rate of recovery [35]. These effects are partly induced by neuron-released Nrg1 promoting Schwann cell differentiation, proliferation, migration, and myelination [28, 36–41].

### Electrical stimulation

As mentioned, long-term axotomy results in 33% of neurons losing their capacity to extend axons. However, electrical stimulation results in a 34%–50% increase in the number of neurons extending axons [42] and a 2.3-fold increase in the extent of axon sprouting from transected axons [43] while also increasing the speed of axon regeneration [17, 42]. This influence is exerted through various mechanisms, including direct actions on axotomized neurons [17, 44–47]. The influence of electrical stimulation is similar when applied to acute and long-term injured neurons [46, 48].

## Injury-induced extrinsic neuronal changes reduce their capacity to extend axons

### Reduced Schwann cell capacity to support neuron

Schwann cells release the cytokines MCP-1 and LIF [49], which recruit macrophages and convert them from the M1 to the M2 phenotype. These macrophages secrete high levels of cytokines, which promote axonal outgrowth [50]. However, nerve injury deprives Schwann cells of axon contact, causing them to become senescent and stop producing and releasing neurotrophic factors. Schwann cell development of senescence parallels the decrease in the extent of axon regeneration [8]. Thus, long autografts do not induce axon regeneration and recovery because by the time the axons reach the distal end of the autograft, the Schwann cells have become senescent and do not support axon regeneration [8].

Schwann cell senescence is also associated with a reduction in their c-Jun expression [51], loss of their injury-induced repair phenotype [8, 22, 38, 52], and their down-regulation of the genes for factors required for Schwann cells to support axon regeneration and proteins required to myelinate axons [30]. These include S100, p75, GFAP, BDNF, NGF, NT-3, NT-4, CNTF, GDNF, and small molecule trkB agonists.

Schwann cell senescence also leads to their inability to synthesize and release VEGF [53]. VEGF is essential for inducing vascularization and recruiting macrophages [54, 55] to the injury site, where the macrophage normally also releases VEGF [55, 56]. In addition, senescent Schwann cells lose their capacity to phagocytize axon and myelin debris [57], and without its removal, it inhibits axon regeneration [30]. Therefore, maximizing functional recovery requires nerve repairs be performed <3–6 months post-trauma [3, 9, 58].

## Reversing injury-induced extrinsic neuronal changes by reactivating Schwann cells: applying neurotrophic factors and restoring c-Jun

Nerve injury induces Schwann cell up-regulation of Shh, which induces c-Jun expression [59–61], which leads to c-Jun enhancing axon regeneration through autografts and *in vitro* [62]. However, with prolonged denervation and aging, c-Jun expression decreases in Schwann cells, which is associated with decreased axon regeneration [51]. Nevertheless, axon regeneration can be promoted by reactivating senescent Schwann cells by applying neurotrophic factors, which restores normal levels of Shh and c-June expression [63].

### Reactivating Schwann cells: applying electrical stimulation

Electrical stimulation reactivates Senescent Schwan cells. This induces their expression of P0, Par-3, BDNF, NGF, and GDNF, which initiate and enhance axon regeneration and myelination [64–66].

## PRP promotes axon regeneration

### Platelet-released factors

Platelets contain and release an evolutionarily complex cocktail of factors, including high levels of neurotrophic and other growth factors, such as IL-10, insulin-like growth factors 1 and 2, VEGF [67], BDNF [68], transforming growth factor-β1, HGF, and FGF. This allows platelets to play different essential roles in tissue healing and promoting axon regeneration [69–72].

In animal model studies, PRP significantly enhances the extent of axon regeneration when injected into a nerve following a nerve crush [73], is applied to sites of a nerve crush [74–78], neurorrhaphy [69, 79–82], site of rat prostatectomy [83], following nerve crush, *mycobacterium leprae* (leprosy bacteria) -induced lesion [84], sucrose-induced injury [85], autografts [86, 87], acellular allografts [88], when applied onto or injected into neurorrhaphy sites [89–92], is injected onto injured nerves [89, 93, 94], or short nerve gaps within the preserved epineurium [95], PRP exosomes are injected under the perineurium [96], and site of carpal tunnel syndrome [97–99]. However, questions have been raised about the efficacy of PRP in treating carpal tunnel syndrome [100].

PRP is similarly effective when added to vein grafts [67, 101–104], conduits composed of many different materials [105–111], and when combined with other cells, such as nMSCs [80] applied outside [86] or inside acellular allografts [88], when conduits are composed of platelet gel [112] or platelet-rich fibrin (PRF) [113, 114]. The PRP can induce axon regeneration that is as effective as autologous nerve grafts [112]. It is important to note that when PRP is applied to a rat sciatic nerve crush site, its influence is increased by surrounding the site with a collagen tube [70].

Clinically, bridging nerve gaps with an autograft within a PRP-filled collagen tube [115–118], or only a PRP-filled collagen tube [118], induces meaningful recovery under conditions where allografts alone are ineffective. Thus, platelet-released factors alone can induce axon regeneration.

### PRP-containing leukocytes

Leukocytes are reported to negatively affect axon regeneration by releasing catabolic cytokines and inducing inflammation [119, 120], while leukocyte-poor PRP (LP-PRP) exerts anabolic effects that promote axon regeneration [121, 122]. However, PRP efficacy is reported to increase with increasing leukocytes and white blood cells concentrations, and bioactivity of platelet-released factors. Platelet growth factor concentrations in leukocyte-rich PRP (LR-PRP) depend on the leukocyte concentrations, with the catabolic protease MMP-9 expressed at a considerably high concentration in the LR-PRP [121]. LR-PRP releases significantly more inflammatory mediators, such as TNF-α, IL-6, and IFN-ϒ than LP-PRP. However, it also increases the release of the anti-inflammatory mediators IL-4 and IL-10 [123, 124]. The combination and concentration of PRP platelets, leukocytes, and erythrocytes influence the extent of these factors’ release [120].

A case report showed that LR-PRP induces meaningful recoveries despite long nerve gaps being repaired with a long repair delay, even in an older subject [118]. This influence is greater than that seen in other studies. The better recovery may be because the PRP was prepared using the Zimmer Biomet GPS III centrifuge system, which increases the platelet concentration 9.3-fold and leukocyte concentration 5-fold (Zimmer Biomet Data on File. Validation Report, GPS III Platelet Concentrator, Test new design for GPS III Buoy re-design, OT000183, 2007), which is at least two times higher than in PRP prepared using other devices [125–127].

The influence of PRP is also affected by its concentration of factors, which is influenced by how PRP is prepared [128]. FGF and TGF are rapidly released from platelets, with their concentration decreasing over time, while PDGF and VEGF are released at a constant rate for 7 days [128]. PRP from the Biomet GPS III has the highest concentrations of VEGF and MMP-9 but the lowest TGF concentration [128]. However, it has also been shown that the concentration of cytokines is not directly related to the cellular composition of PRP [128].

### Angiogenesis

Proteomics analysis found that the local application of PRP significantly increases integrin β-8 (ITGB8) expression [95], which promotes angiogenesis [129, 130]. In addition to providing oxygenation to the region of the regenerating axons, Schwann cells use these new blood vessels as their pathway to migrate into the injury site, forming Schwann cell cords that facilitate axon regeneration [55]. Thus, it has been proposed that PRP-released factors contribute significantly to axon regeneration by promoting vascularization, leading to the migration of cells by activating the FAK pathway mediated by integrin β1 [131, 132].

### Limitations of PRP

While many studies show that PRP promotes axon regeneration and recovery, the extent of the efficacy varies greatly. This is unsurprising because no standard techniques exist for preparing or applying PRP. The simplest and least expensive PRP preparation technique is single spin separation, which yields an increased platelet concentration of 2.67-fold [133], while the double spin technique increased it by 2.48 - 5.71-fold, with a mean of 3.47-fold [134]. A PRP 2.5-3.5-fold increased platelet concentration is considerably less effective in rats than a 4.5 - 6.5- or 7.5 - 8.5-fold increase, although both higher concentrations induce similar influences [135].

Working with New Zealand white rabbit 5 mm nerve gaps, PRP with a 2.5–3.5-fold increased platelet concentration induces limited axon regeneration, significantly greater with the higher concentration of 4.5–6.5-fold and 7.5–8.5-fold [95]. Although a 5-fold increased platelet concentration is recommended as the minimum to exert a meaningful physiological effect [136], the optimal concentration for maximal analgesia remains unknown.

### pH

Various devices yield PRP with higher acidification than normal blood, reducing it from 7.35 to 6.8–6.5 [137, 138]. This decreases platelet aggregation by >25% [139–141] and reduces platelet sensitivity to thrombin, resulting in decreased platelet activation, which reduces PRP efficacy. Therefore, it is necessary to avoid PRP acidification during its preparation.

### Glucose

Different PRP preparation devices yield PRP with glucose concentrations increased 3- to 6-fold over the starting blood [138]. Increasing PRP glucose concentration increases platelet activation [142]. Therefore, maximizing the efficacy of PRP required avoiding changes in its glucose level.

### Diet and physiology affect PRP efficacy

A patient’s physiology and diet can greatly affect PRP efficacy. Smoking increases platelet aggregation [143], while alcohol consumption decreases platelet activation and aggregation [144] and reduces platelet responses to thrombin [145] and collagen. Diets including isoflavones [146], caffeine [147], quercetin, a flavonoid [148], and anthocyanins [149] reduce platelet aggregation. Conversely, diets of high saturated fats [150], simple carbohydrates [150], or excessive sugar [151] increase platelet aggregation. Platelets in patients with high blood pressure have lower concentrations of factors than platelets of patients with normal blood pressure [152] and have a decreased whole blood platelet count [153].

### Platelet activation methods

The PRP efficacy is influenced by (1) whether its platelets are activated before or when PRP is applied, (2) the timing of platelet release, (3) the ratios of the various platelet released factors, and (4) their level of bioactivity [154]. Therefore, PRP that does not comply with the necessary physiological parameters will not exert maximal effects [155].

## Potential mechanisms by which platelet-released factors increase axon regeneration

Platelets contain more than 300 identified factors [156, 157]. Many of these have been shown to play important roles in promoting axon regeneration and recovery. However, space limitations do now allow a discussion of these factors.

## Conclusion

Over the past 70 years, little progress has been made clinically in increasing the percentage of patients who recover meaningful function following peripheral nerve injuries and repairs. Two significant steps forward are the demonstration that, clinically, electrical stimulation and the application of PRP enhance axon regeneration and the extent of recovery. However, the efficacy of PRP varies greatly, within and between studies, which may result from differences in how the PRP is prepared and applied, as well as the patient’s physiological status. Therefore, to optimize the influence of PRP, it is necessary to develop a standardized PRP preparation and application protocol. However, it is also necessary to determine which of a subject’s physiological properties, such as diet, consumption of drugs, smoking, and alcohol, must be changed to allow PRP to exert its maximal influences.
